# Differentiation of Glioblastoma and Brain Metastases by MRI-Based Oxygen Metabolomic Radiomics and Deep Learning

**DOI:** 10.3390/metabo12121264

**Published:** 2022-12-14

**Authors:** Andreas Stadlbauer, Gertraud Heinz, Franz Marhold, Anke Meyer-Bäse, Oliver Ganslandt, Michael Buchfelder, Stefan Oberndorfer

**Affiliations:** 1 Institute of Medical Radiology, University Clinic St. Pölten, Karl Landsteiner University of Health Sciences, 3100 St. Pölten, Austria; 2 Department of Neurosurgery, Friedrich-Alexander University (FAU) Erlangen-Nürnberg, 91054 Erlangen, Germany; 3 Department of Neurosurgery, University Clinic of St. Pölten, Karl Landsteiner University of Health Sciences, 3100 St. Pölten, Austria; 4 Department of Scientific Computing, Florida State University, 400 Dirac Science Library Tallahassee, Tallahassee, FL 32306, USA; 5 Clinic for Neurosurgery, Katharinenhospital Stuttgart, 70174 Stuttgart, Germany; 6 Department of Neurology, University Clinic of St. Pölten, Karl Landsteiner University of Health Sciences, 3100 St. Pölten, Austria

**Keywords:** brain tumors, oxygen metabolism, deep learning, MRI, neurooncology, 1D convolutional neural network, glioblastoma, brain metastasis, artificial intelligence

## Abstract

Glioblastoma (GB) and brain metastasis (BM) are the most frequent types of brain tumors in adults. Their therapeutic management is quite different and a quick and reliable initial characterization has a significant impact on clinical outcomes. However, the differentiation of GB and BM remains a major challenge in today’s clinical neurooncology due to their very similar appearance in conventional magnetic resonance imaging (MRI). Novel metabolic neuroimaging has proven useful for improving diagnostic performance but requires artificial intelligence for implementation in clinical routines. Here; we investigated whether the combination of radiomic features from MR-based oxygen metabolism (“oxygen metabolic radiomics”) and deep convolutional neural networks (CNNs) can support reliably pre-therapeutic differentiation of GB and BM in a clinical setting. A self-developed one-dimensional CNN combined with radiomic features from the cerebral metabolic rate of oxygen (CMRO_2_) was clearly superior to human reading in all parameters for classification performance. The radiomic features for tissue oxygen saturation (mitoPO_2_; i.e., tissue hypoxia) also showed better diagnostic performance compared to the radiologists. Interestingly, both the mean and median values for quantitative CMRO_2_ and mitoPO_2_ values did not differ significantly between GB and BM. This demonstrates that the combination of radiomic features and DL algorithms is more efficient for class differentiation than the comparison of mean or median values. Oxygen metabolic radiomics and deep neural networks provide insights into brain tumor phenotype that may have important diagnostic implications and helpful in clinical routine diagnosis.

## 1. Introduction

Glioblastoma (GB, WHO grade 4) and solitary brain metastasis (BM) are the most frequent types of brain tumors in adults [1]. GB is the most common and most lethal primary brain tumor (i.e., it originates from the brain tissue itself) with an incidence of 4 to 5 per 100,000 people per year and a median overall survival time of only 60 weeks [2,3]. It is highly aggressive and rapidly progressing. A BM is found in 10–30% of patients with metastatic cancer at another location in the body. In 55% of these patients, a newly diagnosed solitary BM is the first manifestation of disease with no known history of malignancy at the time of diagnosis [4] making a classification of the brain tumor as BM often very challenging. BM is also associated with a dismal prognosis and a median survival of less than 2 months in patients with untreated BM [5].

The clinical and therapeutic management of GB and BM is quite different and a quick and reliable decision has a significant impact on the clinical outcome. The current gold standard for the management of newly diagnosed GB involves primary surgery with the goal of achieving the maximum possible reduction in tumor volume followed by combined adjuvant treatment with fractioned stereotactic radiation and concomitant oral chemotherapy with temozolomide [6]. On the other hand, in the case of suspected BM, CT staging is required first to search for a possible primary tumor and assess the extent of metastatic disease. Consideration of surgical management of BM depends on the number and location of the lesions as well as the stage of the primary disease. Stereotactic radiosurgery is considered an effective strategy in the treatment of BM with the advantage of excellent local control rates with minimal invasiveness [7]. Consequently, accurate and reliable initial classification of these two brain tumor entities is critical for personalized decision-making and treatment planning [8].

Magnetic resonance imaging (MRI) is the gold standard in clinical neuroradiology of brain tumors due to its excellent soft tissue contrast. However, differentiation of GB and BM is still challenging due to their very similar appearance as hyperintense brain lesions on contrast-enhanced T_1_-weighted MRI surrounded by hyperintense edema on T_2_-weighted MRI [9,10]. In order to overcome the disadvantage of the low specificity of clinical routine MRI, a variety of innovative imaging methods have been developed and evaluated in recent decades. Indeed, methods for blood perfusion imaging such as arterial spin labeling (ASL) or dynamic contrast-enhanced perfusion MRI, also include methods for metabolic or molecular imaging such as chemical exchange saturation transfer (CEST) MRI, MR spectroscopy, hyperpolarized MRI, or positron emission tomography (PET) using radiolabeled amino acids such as ^11^C-methyl-methionine (MET) or ^18^F-fluoroethyl-tyrosine (FET) to name just a few. The important relevance of tissue hypoxia in the pathophysiology of tumors and the consideration of reprogrammed energy metabolism as a hallmark of cancer [11], i.e., the switch from oxidative phosphorylation to aerobic glycolysis, also known as the Warburg effect, was our motivation to introduce an MR-based approach for the quantitative characterization of oxygen metabolism and tissue hypoxia in primary and secondary brain tumors, respectively [12,13,14,15,16,17]. These multiparametric metabolic imaging data reflect complex pathophysiological interactions and require a time- and labor-intensive evaluation by specially trained personnel, making implementation into the clinical routine hardly feasible without significant computer support.

Radiomics is an emerging field which applies advanced computational approaches to convert medical images into quantitative features [18,19,20]. These radiomic features are able to provide information about the gray-level patterns and their associations within a volume of interest that are not recognizable with the naked human eye. As a result, radiomics offers the possibility of providing additional valuable information for tumor characterization and personalized therapy management, which are obtained directly from noninvasive medical imaging data and not from tissue samples. Hundreds of texture and histogram-based parameters can be extracted from a single imaging data set, further increasing data volume and making it unmanageable for the decision-making clinician.

Artificial intelligence (AI) methods such as deep learning (DL) and traditional machine learning (ML) enable the assessment of a variety of imaging parameters through the generation of multiparametric models. This opens up both the possibility of dealing with large amounts of data and increasing the comparability of the results achieved, since this is independent of the level of experience of the evaluating clinician. Deep learning methods demonstrated the ability to learn differences between classes (different brain tumor entities in our case) from feature representations to be analyzed. These differences in features are “extracted” in hidden layers of neural networks [21]. Convolutional neural networks (CNN) for instance have been demonstrated to have good prediction performances in recognizing simple patterns in data, which are then used to form more complex patterns within deeper layers [22]. In particular, one-dimensional CNNs have proven to be very effective when we have to derive relevant features from fixed-length segments such as radiomic feature vectors [23].

Based on these considerations, we propose in this paper a DL-based method to assess radiomic feature vectors from MR-based oxygen metabolism data to distinguish between GB and BM pre-therapeutically. This is intended to support radiologists and clinicians in timely diagnosis and personalized treatment decisions. Our hypothesis was that the combination of the superior information extraction capability of MRI-based oxygen metabolomic radiomics and the highly effective data analysis of 1D-CNNs has the potential to uncover disease characteristics that are useful for reliable differentiation of GB and BM in a clinical setting.

## 2. Materials and Methods

### 2.1. Ethics

The study was approved and publicly registered by the Ethics Committee of the Lower Austrian Provincial Government (protocol code GS1-EK-4/339-2015, date of approval: 29 February 2016). The study was conducted in accordance with the guidelines of the Declaration of Helsinki. All included patients provided written informed consent prior to enrolment.

### 2.2. Patient

Patients with untreated contrast-enhancing brain tumors that were newly diagnosed between January 2016 and April 2022 were selected from a prospectively populated institutional brain MRI database. Inclusion criteria were: (i) age ≥ 18 years; (ii) histopathological confirmation of either glioblastoma (GBM, WHO grade 4) or brain metastasis; (iii) no previous treatment of the brain tumor; (iv) MRI examinations with the study protocol; and (v) clinical routine MRI data were evaluated by at least two board-certified radiologists in consensus.

### 2.3. MRI Data Acquisition

MRI data acquisition was conducted on a clinical 3 Tesla scanner (Trio, Siemens, Erlangen, Germany) equipped with a standard 12-channel head coil. The MRI protocol for clinical routine diagnosis of brain tumors included an axial fluid-attenuated inversion-recovery (FLAIR) sequence, a high-resolution contrast-enhanced T_1_-weighted (CE T1w) sequence, an axial diffusion-weighted imaging (DWI) sequence, and a gradient echo dynamic susceptibility contrast (GE-DSC) perfusion MRI sequence that was performed using 60 dynamic measurements during the administration of 0.1 mmol/kg-bodyweight gadoterate-meglumine (Dotarem, Guerbet, Aulnay-Sous-Bois, Paris, France).

The MRI-based assessment of tissue oxygen metabolism using the quantitative blood-oxygen-level-depended (qBOLD) imaging approach [12,14] additionally required a multi-echo GE sequence and a multi-echo spin echo (SE) sequence for mapping of the transverse relaxation rates R_2_* (=1/T_2_*) and R_2_ (=1/T_2_), respectively. These MRI sequences were carried out with identical geometric parameters (voxel size, number of slices, etc.) and slice position as used for the GE-DSC perfusion and DWI sequence of the clinical routine protocol. The qBOLD sequences required in total of five minutes of extra scan time.

### 2.4. Calculation of MRI Biomarker Maps of Oxygen Metabolism

Processing of qBOLD data and calculation of MRI biomarker maps for oxygen metabolism was performed with custom-made MatLab (MathWorks, Natick, MA, USA) software. This included the calculation of absolute cerebral blood volume (CBV) and flow (CBF) maps from the GE-DSC perfusion MRI data via automatic identification of arterial input functions (AIFs) [24,25]. R_2_*-mapping raw data required corrections for background fields [26] and R_2_-mapping raw data for stimulated echoes [27], respectively, which was followed by the calculation of R_2_*- and R_2_-maps. For the calculation of biomarker maps of tissue oxygen metabolism including oxygen extraction fraction (OEF) and cerebral metabolic rate of oxygen (CMRO_2_) [28], the following equations were used:

(1) $$\mathrm{OEF}=\frac{R_{2}^{*}-{\;R}_{2}}{\frac{4}{3}{\;\times \;\pi \;\times \;\gamma \;\times \;\Delta \chi \;\times \;\mathrm{Hct}\;\times \;B}_{0\;}\times \;\mathrm{CBV}}$$

(2) $${\mathrm{CMRO}}_{2}=\frac{C_{a}\;\times \;\mathrm{CBF}}{\frac{4}{3}{\;\times \;\pi \;\times \;\gamma \;\times \;\Delta \chi \;\times \;\mathrm{Hct}\;\times \;B}_{0}\;\times \;\mathrm{CBV}}\;\times \;\left(R_{2}^{*}-{\;R}_{2}\right)$$

where R_2_* and R_2_ are the transverse relaxation rates calculated as described above, γ = 2.67502 × 10^8^ rad/s/T is the nuclear gyromagnetic ratio; Δχ = 0.264 × 10^−6^ is the difference between the magnetic susceptibilities of fully oxygenated and fully deoxygenated hemoglobin; Hct = 0.42 × 0.85 is the microvascular hematocrit fraction, whereby the factor 0.85 stands for a correction factor of systemic Hct for small vessels, B_0_ is the main static magnetic field of the MRI scanner, and Ca = 8.68 mmol/mL is the arterial blood oxygen content [29]. Maps of capillary oxygen tension (capiPO_2_) and mitochondrial oxygen tension (mitoPO_2_) [28,30] were calculated with

(3) $${\mathrm{capiPO}}_{2}{=P}_{50}\times \sqrt[{h}]{\left(\frac{2}{\mathrm{OEF}}-\;1\right)}$$

(4) $${\mathrm{mitoPO}}_{2}{=P}_{50}\times \sqrt[{h}]{\left(\frac{2}{\mathrm{OEF}}-\;1\right)}-\frac{{\mathrm{CMRO}}_{2}}{L}$$

where P_50_ is the hemoglobin half-saturation tension of oxygen (27 mmHg), h is the Hill coefficient of oxygen binding to hemoglobin (2.7), and L (4.4 mmol/Hg per minute) is the tissue oxygen conductivity as defined by Vafaee and Gjedde [31].

### 2.5. 3D Radiomic Feature Extraction of Oxygen Metabolism

For a patient, the CE T1w MRI data and the biomarker maps for oxygen metabolism were loaded into the open-source software platform 3D Slicer (v. 4.11) for coregistration. In the first step, the tumor volume was segmented as the contrast-enhancing area on the CE T1w MRI data [32] by a radiologist (G.H., with 30 years of experience in neuro-oncological imaging) and a neurosurgeon (F.M., with 15 years of experience) in consensus. Both readers were blinded to the histopathological diagnosis of the tumor.

In the next step, discretization of the biomarker values was performed in order to obtain histograms with fixed bin numbers using adapted bin width values. We followed the recommendations of the Imaging Biomarker Standardization Initiative (IBSI) [33] and the findings of a previous study [34] that a bin number between 60 and 80 was optimal for the classification of pediatric posterior fossa tumors using metrics and textural features of ADC maps. We chose a bin number of 67 as this gave an integer bin size for most maps. Maps of oxygen metabolism have a range of physiological reasonable values allowing for the application of individually adapted thresholds to remove non-physiological values due to imaging artefacts (e.g., motion or susceptibility artefacts). The ranges of values and bin sizes were for OEF: 0–100%, bin size 1.5; for CMRO_2_: 0–1000 µmol/100 g × min, bin size 15; for capiPO_2_ and mitoPO_2_: 0–200 mmHg, bin size 3. Finally, the 3D Slicer built-in package SlicerRadiomics, which is based on the Python package PyRadiomics [35], was used for radiomic feature extraction. All procedures and features were in accordance with the Imaging Biomarker Standardization Initiative (IBSI) [33].

For 3D radiomic feature extraction of oxygen metabolism within the manually segmented tumor volume, we calculated 14 shape features representing the three-dimensional size and shape of the segmented contrast-enhancing tumor volume in the maps of oxygen metabolism, 18 first-order features that evaluate the distribution of the values for oxygen metabolism within the histogram of the tumor volume, and 75 texture features describing relationships between neighboring voxels with similar or dissimilar values. The latter included five subcategories: (i) 24 gray-level co-occurrence matrix (GLCM) features characterizing how often pairs of voxels with specific intensity levels and spatial relationship occur in an image [36]; (ii) 14 gray-level dependence matrix (GLDM) features representing the dependency of connected voxels to a center voxel [37]; (iii) 16 gray-level run-length matrix (GLRLM) features evaluating the length of consecutive pixels with the same gray level [38]; (iv) 16 gray-level size zone matrix (GLSZM) features quantifying the number of connected voxels that share the same intensity value [39]; and (v) five neighboring gray-tone difference matrix (NGTDM) features assessing differences between pixel values and neighbor average gray value [40].

**Figure 1:**
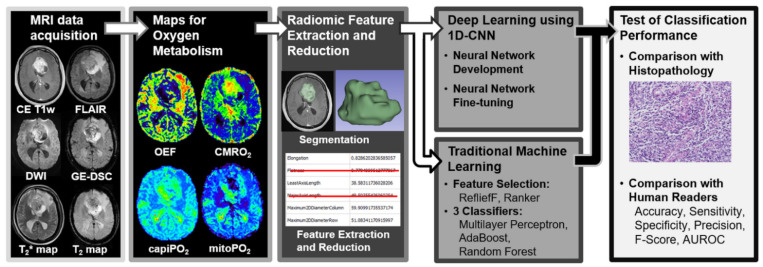
General diagram of the proposed radiomics and radiophysiomics approach, showing the major steps: MRI data acquisition, calculation of MRI biomarker maps of oxygen metabolism, extraction of radiomic feature for oxygen metabolism in the tumor volume and feature reduction, development of a 1D-CNN and traditional ML algorithms for brain tumor classification, and testing of the classification performance.

A detailed overview and description of these radiomic features can be found elsewhere [35,41]. Mathematical formulas have been described on the website of the package (https://pyradiomics.readthedocs.io; accessed on 20 October 2022). This procedure resulted in 107 features describing the spatial properties of the oxygen metabolism across the tumor volume. In order to exclude features with low stability against perturbations in tumor segmentation, another coauthor (A.S., medical physicist with 23 years of experience in brain cancer imaging) manually segmented the contrast-enhancing area on the CE T1w MRI data in 50 randomly sampled patients. The intra-class correlation coefficient (ICC) was calculated for each radiomic feature as described previously [42] using SPSS (version 21, IBM, Chicago, IL, USA). Only features with high reproducibility (ICC ≥ 0.8) were included in the further analysis [42]. Radiomic features with ICC values below this threshold were discarded from further analysis, as shown previously [43]. Due to the marked difference in the number of patients suffering from GBM and brain metastasis, respectively, the synthetic minority oversampling technique (SMOTE) [44] was employed in order to balance the classes. The overall study pipeline is shown in Figure 1.

### 2.6. Deep Learning

**Figure 2:**
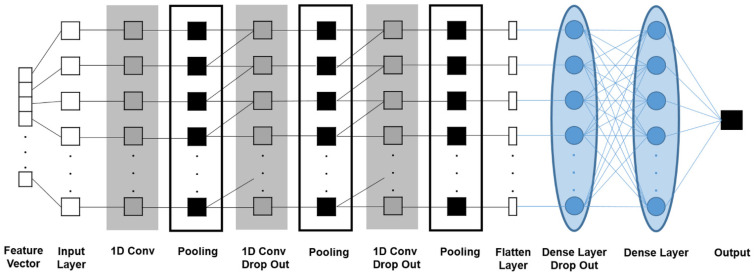
Architecture of the one-dimensional convolutional neural network (1D-CNN).

The open-source data analytics platform KNIME (Konstanz Information Miner; version 4.6.1) was used as it is widely known and gives good performance in biomedical and health engineering applications [45,46,47]. The data were partitioned into training data sets (70% of the patients), validation (15% of the patients), and test data sets (15% of the patients), respectively. For all data partitions, z-score normalization was performed. Convolutional neural networks (CNNs) are the most widely used architectures in deep learning approaches that are generally composed of an input layer, several hidden layers (convolution, pooling, and fully connected “dense” layers), and an output layer. The deep neural network developed for brain tumor classification was based on a one-dimensional CNN (1D-CNN) [48] characterized by the fact that during convolution the CNN kernels slide only over the elements of one dimension of the input pattern, here the vector of oxygen metabolic features. The architecture included nine hidden layers: three convolutional layers, three max-pooling layers, one flattened layer, and two fully connected (dense) layers. The activation function used a rectified linear unit (ReLU) for all convolutional layers and the first dense layer [49]. The final dense layer, however, applied a sigmoid function for activation and output with one unit for binary classification (GB or BM). Three dropout rates of 0.2 were used after the second and third convolution layer as well as after the first dense layer, respectively, in order to avoid overfitting [50]. As a loss function, we consider the binary cross-entropy, while as an optimizer we exploit Adam (adaptive moment estimation), an adaptive learning rate optimization algorithm specifically designed for deep neural networks training. The architecture of the 1D-CNN is depicted in Figure 2 and the parameters are summarized in Table 1. The DL model was trained with 70% and validated with 15% of the patient data. The remaining 15% were used as independent test cohort. The training data were shuffled before each epoch (up to 300) in order to robustness of the models.

### 2.7. Traditional Machine Learning

Development of traditional ML algorithms was carried out for comparison purposes with the open-source software package WEKA (version 3.8.5, University of Waikato, Hamilton, New Zealand). In the first step, radiomic feature selection was performed using the well-known attribute evaluation filter (RefiefF) in combination with the Ranker search method. The top-ranked features (approximately 25% of all features) were selected. The data were partitioned into training/validation data sets (85% of the patients) and test data sets (15% of the patients), respectively, and z-score normalization was performed. Next, the three best-performing ML algorithms for multiclass differentiation between the five contrast-enhancing brain tumor entities GBM, anaplastic glioma, primary CNS lymphoma, meningioma, and brain metastasis, as demonstrated in a previous study [51] were used for binary differentiation between GBM and brain metastasis. These traditional ML algorithms were: multilayer perceptron (MLP; one hidden layer, number of neurons = number of features + number of classes), adaptive boosting (AdaBoost; using decision tree “J48” as a classifier), and random forest (RF). The training/validation data sets for CMRO_2_, OEF, capiPO_2_, and mitoPO_2_ as well as all these parameters of oxygen metabolism together (OxyMet) were used for training the algorithms. A tenfold cross-validation procedure was adopted for the validation of the models. Finally, the trained models were tested with unseen data from the testing data set using confusion matrix-derived metrics and AUROC as described below.

### 2.8. Human Reading

At least two board-certified radiologists reviewed the clinical routine MRI data (i.e., FLAIR, CE T1w, ADC, and CBV) and other anatomical MRI data, which were part of the routine protocol for diagnosis of contrast-enhancing brain tumors. The readers had access to the clinical information of each patient. The readers recorded a final diagnosis in consensus for each patient and the most likely diagnosis was used for assessment of the diagnostic performance of the human readers.

### 2.9. Testing of the Models

The classification performance for both the deep learning model (1D-CNN) and the traditional ML algorithms were tested with unseen data from an independent test cohort of patients (*n* = 34). The main performance evaluation metric was the area under the receiver operating characteristic curve (AUROC) [52]. In addition, confusion matrix-derived metrics including accuracy, sensitivity (aka recall or true positive rate), specificity (aka true negative rate), precision (aka positive predictive value), and the F-score were calculated. The performance metrics for each brain tumor entity were calculated by averaging the ten different validation performances followed by the calculation of the weighted average over the five brain tumor entities.

## 3. Results

### 3.1. Patient Characteristics

The institutional brain MRI database searched for this study contained a total of more than 1800 MR examinations using the study protocol in 600 brain tumor patients. From January 2016 to June 2022, a total of 133 patients (64 females; 69 males; mean age 62.8 ± 12.2 years; 19–84 years) with newly diagnosed, untreated contrast-enhancing brain tumors satisfied the inclusion criteria. Of these, 96 patients (72%; 43 females; 53 males; mean age 62.5 ± 13.4 years; 19–84 years) had the diagnosis of a glioblastoma WHO grade 4 and 37 patients (28%; 21 females; 16 males; mean age 63.6 ± 8.4 years; 46–79 years) suffered from a brain metastasis that originated in fifteen patients from lung cancer, in six patients from breast cancer, in six patients from a melanoma, in two patients each from esophageal, bladder, colon, or renal cancer, and in one patient each from fibrosarcoma or pancreatic cancer, respectively.

### 3.2. The Selected Radiomic Features

From the 107 features that were extracted for each MRI data set and VOI, respectively, 74 features with high reproducibility (ICC ≥ 0.8) were selected: 3 shape features, all 18 first-order features, and 53 texture features (19 GLCM, 9 GLDM, 10 GLRLM, 11 GLSZM, 4 NGTDM). An overview of the selected and excluded features is provided in Table 2.

### 3.3. Performance of the Deep Learning Model

**Figure 3:**
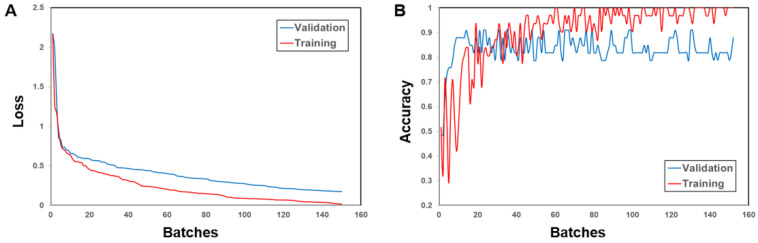
The initial phase of the training process of the one-dimensional convolutional neural network (1D-CNN) is represented by the loss curve (**A**) and the accuracy curve (**B**) with batches. Five batches corresponded to one epoch.

The initial phase of the training process (the first 30 epochs or 150 batches) of the DL model (1D-CNN) is depicted in Figure 3. The loss curve (Figure 3A) demonstrates that the loss showed a sharp drop during the first few batches (five batches per epoch) and tended to be flat as the number of processed batches increases indicating that the training model converges. In the accuracy curve for training (red line in Figure 3B), however, the values showed a strong fluctuation during the first batches followed by a rapid increase to the maximal value (accuracy = 1). The accuracy values for validation (blue line in Figure 3B) increased stronger but the maximum ranged between 0.8 and 0.9, very likely because of the relatively small number of patients in the validation cohort.

**Figure 4:**
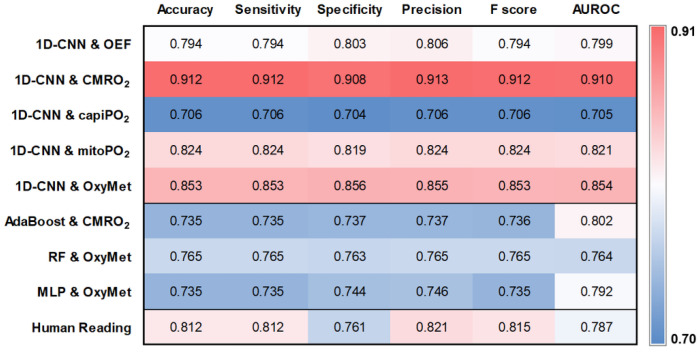
Heat maps depicting (left-right) accuracy, sensitivity, specificity, precision, f-score, and area under the receiver operating characteristic (AUROC) curve for brain tumor classification using a 1D-CNN in combination with radiomics features of oxygen metabolism representing (top-down) oxygen extraction fraction (OEF), the cerebral metabolic rate of oxygen (CMRO_2_), capillary oxygen tension (capiPO_2_), mitochondrial oxygen tension (mitoPO_2_), and all determined measures of oxygen metabolism (OxyMet) together, i.e., OEF, CMRO_2_, capiPO_2_, and mitoPO_2_. Below are the parameters of the diagnostic performance for the top-performing traditional ML algorithms adaptive boosting (AdaBoost), random forest (RF), and multilayer perceptron (MLP), as well as the diagnostic performance for human readers. The color code is on the right-hand side. Note: For both 1D-CNN and traditional ML SMOTE technique was employed to balance the classes that resulted in 192 cases (133 real-world + 59 “synthetized” cases). Performance parameters for human reading, however, were based on the 133 real-world cases only.

For the test cohort of 34 patients, the 1D-CNN showed the best classification performance when combined with the radiomic features from CMRO_2_ with an accuracy of 0.912, precision of 0.913, and AUROC of 0.91, respectively. This was followed by the combination of the 1D-CNN with the radiomic features of all four parameters for oxygen metabolism together (“OxyMet”; accuracy of 0.853, precision of 0.855, and AUROC of 0.854) and with the radiomic features of mitoPO_2_ (i.e., tissue hypoxia; accuracy of 0.824, the precision of 0.824, and AUROC of 0.821), respectively. Interestingly, both the mean and median values for quantitative CMRO_2_ and mitoPO_2_ values did not differ significantly between GB and BM by a Student’s *t*-test: mean CMRO_2_ for GB = 121 µmol/100 g × min vs. mean CMRO_2_ for BM = 123 µmol/100 g × min; P = 0.871; median CMRO_2_ for GB = 85.6 µmol/100 g × min vs. median CMRO_2_ for BM = 90.4 µmol/100 g × min; P = 0.582; and mean PO_2_ for GB = 55.7 mmHg vs. mean PO_2_ for BM = 49.6 mmHg; P = 0.195; median mitoPO_2_ for GB = 48.3 mmHg vs. median PO_2_ for BM = 44.0 mmHg; P = 0.421. This showed the efficiency of the combination of radiomic features and DL algorithms in extracting and utilizing valuable information that is not recognizable as such at first glance. The features for OEF and capiPO_2_ in combination with the DL model showed the worst performance for differentiation between GB and BM. Figure 4 (upper part) provided an overview of all six performance parameters and all five combinations (1D-CNN and oxygen metabolomic radiomic features) such as heat maps.

### 3.4. Comparison with Traditional Machine Learning Models and Human Readers

The values for accuracy, sensitivity, specificity, precision, F-score, and AUROC for the three best-performing combinations of traditional ML models and radiomic features for oxygen metabolism when applied to the test cohort are also presented in the heat map of Figure 4. Almost all parameters were less than 0.8 and thus showed significantly poorer performance than the best-performing DL models.

**Figure 5:**
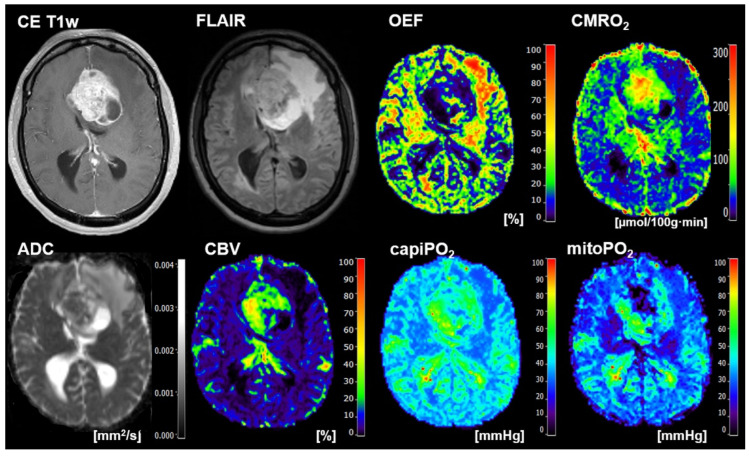
Representative case of a 47-year-old female patient suffering from a brain metastasis from breast cancer that was correctly classified by the 1D-CNN and CMRO_2_ features but misclassified as GB by the radiologists. Contrast-enhanced (CE) T1w and FLAIR MRI data combined with the quantitative maps of apparent diffusion coefficient (ADC) and cerebral blood volume (CBV) among others were used for human reading by two board-certified radiologists in consensus. MRI biomarker maps for oxygen metabolism including oxygen extraction fraction (OEF), the cerebral metabolic rate of oxygen (CMRO_2_), capillary oxygen tension (capiPO_2_), and mitochondrial oxygen tension (mitoPO_2_), respectively, of the patient are presented on the right-hand part of the Figure.

**Figure 6:**
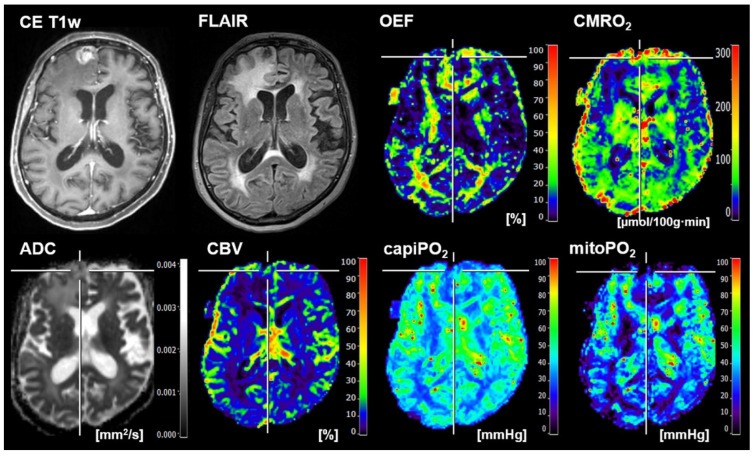
Representative case of a 66-year-old female patient suffering from a brain metastasis from melanoma that was misclassified as GB by the 1D-CNN but correctly classified by the radiologists. Contrast-enhanced (CE) T1w and FLAIR MRI data combined with the quantitative maps of apparent diffusion coefficient (ADC) and cerebral blood volume (CBV) among others were used for human reading by two board-certified radiologists in consensus. MRI biomarker maps for oxygen metabolism including oxygen extraction fraction (OEF), the cerebral metabolic rate of oxygen (CMRO_2_), capillary oxygen tension (capiPO_2_), and mitochondrial oxygen tension (mitoPO_2_), respectively, of the patient, are presented on the right-hand part of the Figure.

The classification results for human reading for all 133 patients are summarized at the bottom of Figure 4. With their values for the performance parameters between 0.761 (specificity) and 0.815 (F-score), the radiologists were in the midfield, between the best DL models and the traditional ML models. The conventional MRI data used by the radiologists to make a diagnosis (left part) and the biomarker maps for oxygen metabolism (right part) for a representative patient suffering from brain metastasis from breast cancer are presented in Figure 5. The patient was correctly classified by the combination of CMRO_2_ features and 1D-CNN but misclassified as GB by the radiologists. In the independent test cohort of thirty-four patients, six patients were misclassified by the radiologists (2 GB and 4 BM) and three patients by the best-performing 1D-CNN (1 GB and 2 BM), respectively. These three patients were also misclassified by the human readers. A case for misclassification by the 1D-CNN only is presented in Figure 6.

## 4. Discussion

Treatment strategies differ significantly between brain cancer entities. Therefore, fast and reliable classification is essential for personalized therapy management, but remains a major challenge in today’s clinical neurooncology. In particular, GB and BM are often difficult to distinguish on conventional MRI scans, since both entities typically appear as well-defined, ring-enhancing lesions with central necrosis on CE T1w MRIs and peritumoral edema as hyperintensity on T2w MRIs [53]. Several studies have revealed diagnostic uncertainties and a limited ability of conventional MRI to distinguish GB from BM [54,55,56]. In the last decades, novel metabolic, molecular, or physiological neuroimaging methods have been developed that have proven useful for improving diagnostic performance [57,58]. However, this requires time-consuming and labor-intensive evaluations by specially trained personnel, which necessitate considerable computer support for implementation in the clinical routine.

In this study, we demonstrated that the combination of radiomic features from MR-based oxygen metabolism (“oxygen metabolic radiomics”) and deep convolutional neural networks can support reliably pre-therapeutic differentiation of GB and BM in a clinical setting. A 1D-CNN combined with radiomic features from CMRO_2_ was clearly superior to human reading in all parameters for classification performance. The combination with the radiomic features for oxygen saturation in the tissue (mitoPO_2_) also showed better diagnostic performance compared to the radiologists. Interestingly, both the mean and median values for quantitative CMRO_2_ and mitoPO_2_ values did not differ significantly between GB and BM. This demonstrates that the combination of radiomic features and DL algorithms is more efficient for class differentiation than the comparison of mean or median values. In addition, the 1D-CNN significantly outperformed traditional ML algorithms. Indeed, the higher complexity of DL algorithms could be related to feature selection, which is a critical step for ML methodology.

A previous study [59] using tumor microenvironment mapping (TME) in contrast-enhancing brain tumors revealed significant differences in aerobic glycolysis, oxidative phosphorylation, and intratumoral tissue hypoxia between GB and BM. It is well known that hypoxic compartments are typical features of the TME of many aggressive cancers, especially GB. High rates of proliferation cause high oxygen demand. Uncontrolled neovascularization leads to intravascular thrombosis, hemorrhage, and dysfunctional vasculature with hypoperfusion, eventually leading to nutrient starvation and tissue hypoxia [60]. Furthermore, to sustain rapid proliferation, cancer cells metabolize glucose to lactate even in the presence of oxygen (i.e., aerobic glycolysis) for both energy production and the generation of carbon molecules essential for cancer biosynthesis, a phenomenon known as the Warburg effect [61]. The intratumoral heterogeneity of necrosis, hypoxia, and neovascularization in combination with the dominant energy metabolic pathway shapes the landscape of the TME [62]. Radiomic features are particularly useful for extracting information from spatial heterogeneous patterns that are not visible to the naked human eye. This was also demonstrated in our study where we were able to distinguish GB and BM with radiomic features extracted from oxygen metabolic mapping with higher diagnostic power compared to radiologists.

AI support for pre-therapeutic diagnostics of brain tumors based on MRI data has attracted great interest in the research community in recent years and has led to a number of publications in the literature. The vast majority of previous studies [63,64,65,66] have used radiomic features from conventional anatomical MRI data in combination with DL and/or ML methods for preoperative differentiation of GB and BM. Taricotti, et al. [67] examined the classification performance of the pre-trained deep learning algorithm ResNet-101 in combination with manually segmented CE T1w MRI scans of 84 patients with GB (47 patients) or BM (37 patients). They achieved an accuracy of 83% and 81% for GB and BM, respectively, and a precision of 76% and 71% for GB and BM, respectively. In another study, Shin et al. [68] used CE T1w and T2w MRI data from 498 patients with GB or solitary BM to perform classification by transfer learning of a pre-trained ResNet-50 model. They achieved accuracy and precision of 89% and 85%, respectively, for an internal test cohort, and 86% and 91%, respectively, for an external test cohort. The performance parameters of an experienced neuroradiologist were 89% for accuracy and 87% for precision. Bae et al. [65] used a self-developed CNN and traditional ML algorithms with handcrafted radiomic features from CE T1w and T2w MRI data. The CNN achieved an accuracy of 89% and the best-performing ML (adaBoost) model of 83% for differentiating GB and BM in an independent test cohort.

The performance parameters of the best-performing algorithms in our current study were higher or similar compared to the cited studies. However, our approach was different. We exclusively used MR-based oxygen metabolism data and a self-developed 1D-CNN for classification. Our aim was to investigate the feasibility and usefulness of distinguishing between GB and BM solely on the basis of differences in radiomic features of oxygen metabolism. We were able to show that the feature differences in the metabolic rate of oxygen (CMRO_2_) show a better diagnostic performance without including information of contrast agent uptake or T2w signal changes in peritumoral edema, which is common in clinical practice.

To the best of our knowledge, Baazaoui et al. [69] is the only study so far that used MR-based oxygen metabolism in combination with traditional ML methods to differentiate GB and BM. Their performance parameters for OEF in contrast-enhancing tumors were very similar to our results for OEF: accuracy, 81%; sensitivity, 87%; specificity 75%; and AUROC, 0.79. However, those for CMRO_2_ were significantly lower compared to our results. They achieved the highest diagnostic power by combining OEF in contrast-enhancing tumor and the ratio for the CMRO_2_ values contrast-enhancing tumor/peritumoral non-enhancing T2w hyperintense region evaluated by using a support-vector machine (AUROC, 0.94). However, the authors only included data from 15 patients (7 GB and 8 BM), and the classification performance was not evaluated in an independent test cohort. This must be taken into account when interpreting their results. However, the study already shows the potential of MRI biomarkers of oxygen metabolism for the AI-supported differentiation of GB and BM.

Few studies have used metabolic neuroimaging techniques in combination with DL algorithms for pre-therapeutic classification of brain tumors. Sartoretti et al. [70] investigated the utility of Amide Proton Transfer weighted (APTw) CEST MRI in distinguishing between low- and high-grade gliomas (WHO grade 2–4) and BM. However, they used only traditional ML methods and tenfold cross-validation in 48 patients, but performed no validation in an independent test cohort. A multilayer perceptron provided the best diagnostic performance with a sensitivity of 81.3%, a specificity of 81.1%, and an AUROC of 0.836, respectively. These values are slightly better than those we found for the MLP combined with radiomic features for oxygen metabolism, albeit for an independent test cohort.

Cao et al. [71] investigated the ability of radiomic features from both anatomical MRI and 18F-fluorodeoxyglucose (^18^F-FDG) PET data in combination with multiple ML models to distinguish GB from solitary BM. They analyzed the imaging data of 100 patients (50 GB and 50 BM), but again an independent test cohort was not evaluated. They found that models combining MRI and ^18^F-FDG PET data using joint voting prediction performed better compared to isolated data sets (MRI or ^18^F-FDG PET data alone). A logistic regression model yielded an accuracy of 89% and an AUROC of 0.91.

In an interesting paper from N. Dikaios [72] ^1^H-MRS data from multiple institutions were used in combination with 1D-CNNs to differentiate GB from BM. Like the MRI-based determination of oxygen metabolism, MRS is rarely included in clinical routine MRI protocols. As a result, training data for DL methods are usually not available in sufficient quantity and/or were acquired on different MR scanners with different data acquisition techniques. To overcome this limitation, the author proposed a novel method to quantum-mechanically synthesize MRS data for any data acquisition protocol. The DL algorithms were trained on the augmented synthetic spectra and tested on two independent data sets acquired from different scanners. By training the 1D-CNNs with several thousand augmented synthetic spectra, the accuracy increased from 77–83% to 88–93% and the AUROC increased from 0.84–0.88 to 0.94–0.97. This approach of increasing the amount of training data through data synthesis is also interesting for the method used in this study but requires the development of suitable data synthesis models.

Besides the relatively small number of patients (133 patients) and the imbalance of the data (96 GB and 37 BM), which could possibly be addressed with synthetic data, we would like to point out some further limitations of our present study. This was a single-center study and data acquisition was carried out with a single MR scanner. This study design did not take into account variations in examination protocols at different magnetic field strengths with different MR scanner setups. Although physiological MRI of oxygen metabolism is still very rarely used in clinical routine, we plan a multicenter study to test its applicability in a larger clinical setting and to generate more data. Data pre-processing and radiomic feature extraction were carried out manually. This is very time-consuming and labor-intensive, which reduces compatibility with clinical routine and also reduces the number of patients that can be enrolled. Therefore, the implementation of a user-independent DL-based approach for automated brain tumor segmentation and feature extraction will be the first next step, especially in the preparation of the multi-center study. On the other hand, handcrafted radiomic features have the advantage of assessing the entire tumor volume, while 2D-CNNs only process a single 2D image slice. Therefore, a 3D-CNN is required for this purpose. Finally, we only performed binary classification, which requires patient selection before classification. With only two possible classes, the error probability is lower than with multiclass classification, which is closer to clinical reality. Other contrast-enhancing brain tumor entities such as meningiomas, primary CNS lymphoma, or ependymomas, as well as contrast-enhancing non-tumorous processes, e.g., brain abscesses were not included. For these pathologies, the patient numbers (<5–15) were too small for deep learning.

## 5. Conclusions

In this paper, we examined the feasibility and usefulness of radiomic features from MRI-based oxygen metabolism (oxygen metabolic radiomics) in combination with a DL algorithm (a 1D-CNN) for pre-therapeutic differentiation between GB and BM, the most common and difficult-to-distinguish types of contrast-enhancing brain tumors in adults. Although mean and median values for CMRO_2_ and PO_2_ showed no significant differences between the two tumor entities, the radiomic features of these oxygen-metabolic biomarkers were particularly well suited for classification using DL. In our opinion, this clearly demonstrated the greater information content of radiomic features. However, due to the complexity and lack of intuitiveness of the radiomic features, an AI-based evaluation is required. On this topic, the superiority of DL-based algorithms over traditional ML algorithms could be clearly demonstrated. In summary, oxygen metabolic radiomics and deep neural networks provide insights into tumor phenotype that may have important diagnostic implications. This approach could be helpful in clinical routine diagnosis, however, incorporation of deep neural networks for radiomic feature extraction, in combination with radiomic features extracted from other MRI techniques, and multicenter studies are required in the near future. However, the direct application of CNNs to the metabolic maps requires the inclusion of e.g., the Gradient-weighted Class Activation Mapping (Grad-CAM) technique [73] for producing visual explanations of CNN-based decisions and overcoming the black-box limitation of 2D- and 3D-CNNs. In addition, the application for therapy monitoring and recurrence detection is also a next logical step with high clinical relevance.

## Figures and Tables

**Table 1:** Parameters of one-dimensional convolutional neural network (1D-CNN).

Type	Input Size ^1^	No. of Filters	Kernel Size	Stride	Padding	Activation
Input	74, 1 (296, 1)					
1D Convolution Layer	74, 1 (296, 1)	8	2	1	same	ReLU ^2^
Max Pooling Layer	74, 8 (296, 8)		2	1	same	
1D Convolution Layer	74, 8 (296, 8)	8	3	1	same	ReLU
Drop out (rate 0.2)	74, 8 (296, 8)					
Max Pooling Layer	74, 8 (296, 8)		2	1	same	
1D Convolution Layer	74, 8 (296, 8)	8	3	1	same	ReLU
Drop out (rate 0.2)	74, 8 (296, 8)					
Max Pooling Layer	74, 8 (296, 8)		2	2	same	
Flatten Layer	37, 8 (148, 8)					
Dense layer	296 (1184)					ReLU
Drop out (rate 0.2)	296 (1184)					
Dense layer	296 (1184)					Sigmoid

^1^ Tensor sizes for application to all four maps of oxygen metabolism together (“OxyMet”) are given in parentheses. ^2^ ReLU = rectified linear unit.

**Table 2:** Selected and excluded features based on reproducibility (ICC ≥ 0.8).

	Features with High Reproducibility (ICC ≥ 0.8)	Excluded Features
Shape Features	Elongation, Mesh Volume, Voxel Volume	Flatness, Least Axis Length, Major Axis Length, Maximum 2D Diameter Column, Maximum 2D Diameter Row, Maximum 2D Diameter Slice, Maximum 3D Diameter, Minor Axis Length, Sphericity, Surface Area, Surface Volume Ratio
First-Order Features	10th Percentile, 90th Percentile, Energy, Entropy, Interquartile Range, Kurtosis, Maximum, Mean Absolute Deviation, Mean, Median, Minimum, Range, Robust Mean Absolute Deviation, Root Mean Squared, Skewness, Total Energy, Uniformity, Variance	
Texture Features	GLCM ^1^: Autocorrelation, Cluster Prominence, Cluster Shade, Cluster Tendency, Contrast, Correlation, Difference Average, Difference Entropy, Difference Variance, Imc1, Imc2, Inverse Variance, Joint Average, Joint Energy, Joint Entropy, Maximum Probability, Sum Average, Sum Entropy, Sum Squares GLDM ^2^: Dependence Entropy, Dependence Non-Uniformity, Dependence Variance, Gray Level Variance, High Gray Level Emphasis, Large Dependence Emphasis, Large Dependence Low Gray Level Emphasis, Small Dependence Emphasis, Small Dependence High Gray Level Emphasis GLRLM ^3^: Gray Level Non-Uniformity Normalized, Gray Level Variance, High Gray Level Run Emphasis, Long Run Emphasis, Long Run High Gray Level Emphasis, Long Run Low Gray Level Emphasis, Run Entropy, Run Length Non-Uniformity, Run Variance, Short Run High Gray Level Emphasis GLSZM ^4^: Gray Level Non-Uniformity, Gray Level Non -Uniformity Normalized, Gray Level Variance, High Gray Level Zone Emphasis, Large Area Low Gray Level Emphasis, Size Zone Non-Uniformity, Size Zone Non-Uniformity Normalized, Small Area Emphasis, Small Area High Gray Level Emphasis, Zone Entropy, Zone Percentage NGTDM ^5^: Busyness, Complexity, Contrast, Strength	GLCM: Id, ldm, Idmn, Idn, MCC GLDM: Dependence Non-Uniformity Normalized, GLDM Gray Level Non- Uniformity, Large Dependence High Gray Level Emphasis, Low Gray Level Emphasis, Small Dependence Low Gray Level Emphasis GLRLM: Gray Level Non-Uniformity, Low Gray Level Run Emphasis, Run Length Non-Uniformity Normalized, Run Percentage, Short Run Emphasis, Short Run Low Gray Level Emphasis GLSZM: Large Area Emphasis, Large Area High Gray Level Emphasis, Low Gray Level Zone Emphasis, Small Area Low Gray Level Emphasis, Zone Variance NGTDM: Coarseness

^1^ GLCM gray level co-occurrence matrix, ^2^ GLDM = Gray Level Dependence Matrix, ^3^ GLRLM = Gray Level Run Length Matrix, ^4^ GLSZM = Gray Level Size Zone Matrix, ^5^ NGTDM = Neighboring Gray Tone Difference Matrix.

## Data Availability

Data are available on request due to privacy and ethical restrictions.
